# Molecular Phylogeny of the Cyrtophorid Ciliates (Protozoa, Ciliophora, Phyllopharyngea)

**DOI:** 10.1371/journal.pone.0033198

**Published:** 2012-03-12

**Authors:** Shan Gao, Jie Huang, Jiamei Li, Weibo Song

**Affiliations:** Laboratory of Protozoology, Institute of Evolution & Marine Biodiversity, Ocean University of China, Qingdao, China; Biodiversity Insitute of Ontario - University of Guelph, Canada

## Abstract

Evolutionary relationships of cyrtophorian ciliates are poorly known because molecular data of most groups within this subclass are lacking. In the present work, the SS rRNA genes belonging to 17 genera, 7 families of Cyrtophoria were sequenced and phylogenetic trees were constructed to assess their inter-generic relationships. The results indicated: (1) the assignment of cyrtophorians into two orders is consistently confirmed in all topologies; (2) the order Dysteriida is an outlined monophyletic assemblage while Chlamydodontida is paraphyletic with three separate monophyletic families; (3) *Microxysma*, which is currently assigned within the family Hartmannulidae, should be transferred to the family Dysteriidae; (4) the systematic position of Plesiotrichopidae remains unclear, yet the two genera that were placed in this family before, *Pithites* and *Trochochilodon*, should be transferred to Chlamydodontida; (5) a new family, Pithitidae n. fam., based on the type genus *Pithites* was suggested; and (6) the sequence of *Isochona* sp., the only available data of Chonotrichia so far, is probably from a misidentified species. In addition, three group I introns of SS rRNA gene were discovered in *Aegyriana oliva*, among which Aol.S516 is the first IE group intron reported in ciliates.

## Introduction

In the system presented by Lynn [1], the subclass Cyrtophoria, a highly divergent ciliate group, embraces 2 orders, 9 families and 46 genera [1]–[6]. Most schemes depicted this group as a well defined monophyletic assemblage. However, they differ from each other with respect to the relationships and systematic positions among constitute genera, because relatively few morphogenetic criteria can be used in the taxonomy and systematic analyses [7]–[11].

Compared to the huge number of morphotypes recognized to date, molecular information of Cyrtophoria is relatively rare. For example, only 6 cyrtophorian genera have available SS rRNA sequences in the GenBank database, and there were very few molecular investigations performed concerning the phylogeny of this group, but see [12]–[17]. Among them, Snoeyenbos-West et al. [13] provided the molecular support for the monophyly of cyrtophorians for the first time, which was again confirmed by Li & Song [15], [16]. Nevertheless, the above studies generally focused on the relationship of the higher level taxa based on a very limited species selection, while the systematic arrangements among lower-level groups where most confusions and disputes reside have not been clarified [15], [16].

In the current work, we sequenced the SS rRNA gene of 18 species representing 17 genera and subsequently carried out phylogenetic analyses. Our aims are to expand the understanding of the phylogeny of this extremely confusing group, especially focusing on the relationships among genera/families and to supply additional molecular information for future studies on this assemblage.

## Materials and Methods

### Source of organisms and morphological identification

**Figure 1:**
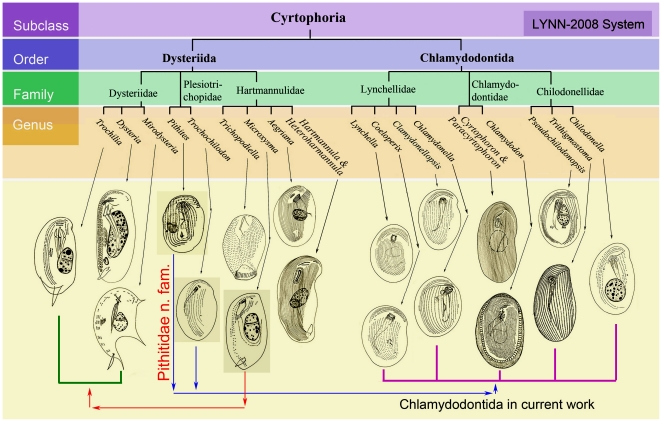
Schematic diagrams of the morphospecies representing genera sequenced in the present study [19]. The cladogram is according to the classification system of Lynn [1]. Arrows indicate the transfer of several species: *Microxysma* from Hartmannulidae to Dysteriidae; *Pithites* and *Trochochilodon* from Dysteriida to Hartmannulida.

Species sequenced in the present study were collected from northern and southern China (Fig. 1, Table S2). Culturing and morphological examination of these species were according to Pan et al. [18]. Species identification was based on the literatures [8], [19], [20]. Terminology and systematic scheme follow Lynn [1].

### DNA extraction, PCR amplification, and sequencing

Cell isolation and genomic DNA extraction were according to Gong et al. [21]. Primers used in the present study were EukA and EukB [22]. The polymerase chain reaction (PCR) followed the protocol of Yi & Song [23].

### Secondary structure of intron

Three introns in the SS rRNA sequence of *Aegyriana oliva* were identified by the alignment of several intron-less cyrtophorian ciliates using CLUSTAL W 1.83 [24]. The secondary structure of introns were predicted by the Group I Intron Sequence and Structure Database (GISSD) [25] by using the covariance model (CM) of the seed alignment of IC1 and IE introns in the package INFERNAL V0.81 (http://infernal.janelia.org/).

### Phylogenetic analyses

**Figure 2:**
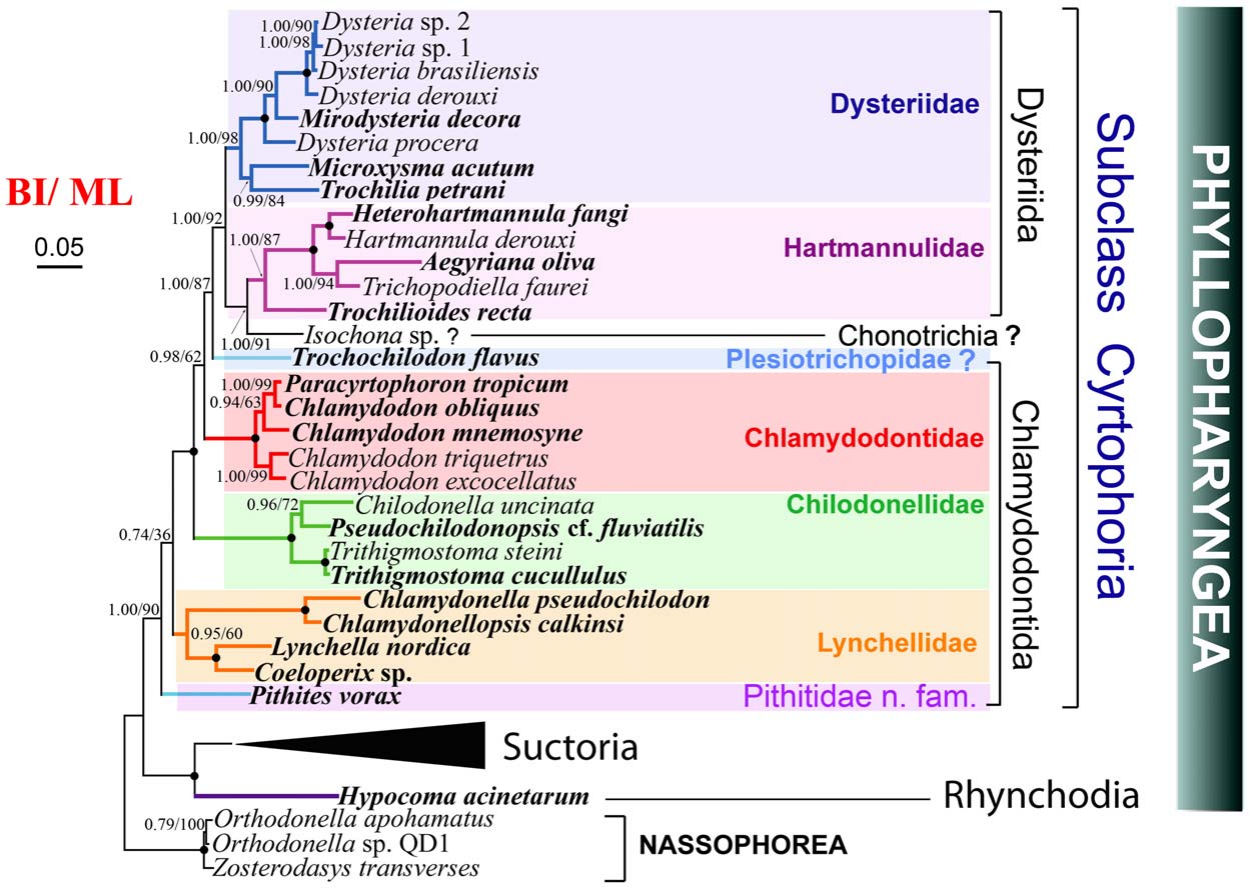
Phylogenetic trees (BI/ML) derived from the dataset 2 of small subunit ribosomal RNA genes. Species newly sequenced in the present study are marked in bold. Numbers at the nodes represent the Bayesian posterior probability value and the bootstrap values from maximum likelihood. Solid circles represent full bootstrap support in both algorithms.

Sequences newly acquired in this study were deposited in the GenBank database with the accession numbers listed in Table 1. Other sequences used for phylogenetic tree construction were obtained from the GenBank database (Table 1). Dataset 1 includes representatives from all the Ciliophora classes, and was aligned with the “Ciliophora” model using Hmmer 2.3.2 [26]. Dataset 2 was scaled down to the two classes, Phyllopharygea and Nassophorea, which was aligned with the “Phyllopharyngea” and “Nassophorea” models. The ambiguously aligned sites were refined using Gblocks v.0.91b [27], yielding an alignment of 1557 and 1455 characters for dataset 1 and dataset 2 respectively. Due to the more specific model used for sequence alignment, phylogenetic trees constructed with dataset 2 have the identical topology as those from dataset 1, but with slightly higher bootstrap value/posterior probability (Figs. 2, S1, S2).

**Table 1:** Accession numbers of the species used for the phylogenetic tree construction.

Species name	GenBank Acc.No.	Species name	GenBank Acc.No.
*Acineta* sp.	AY332718	Litonotus paracygnus*	DQ190464
***Aegyriana oliva*** *	FJ998029	*Loxodes striatus*	U24248
*Blepharisma americanum*	M97909	Loxophyllum jini*	EF123708
*Bresslaua vorax*	AF060453	Lynchella nordica*	FJ998036
*Chilodonella uncinata*	AF300281	*Metopus palaeformis*	M86385
*Chlamydodon excocellatus*	AY331790	Microxysma acutum*	FJ870069
Chlamydodon mnemosyne*	FJ998031	Mirodysteria decora*	JN867020
Chlamydodon obliquus*	FJ998030	*Nassula* sp. QD2*	EU286810
*Chlamydodon triquetrus*	AY331794	*Nyctotheroides deslierrae*	AF145353
Chlamydonella pseudochilodon*	FJ998032	*Obertrumia georgiana*	X65149
Chlamydonellopsis calkinsi*	FJ998033	*Orthodonella apohamatus*	DQ232761
***Coeloperix*** ** sp.** *	FJ998034	*Orthodonella* sp. QD1*	EU286809
*Coleps hirtus*	U97109	Paracyrtophoron tropicum*	FJ998035
*Colpoda inflate*	M97908	*Plagiopyla frontata*	Z29440
*Colpodidiidae* sp. HWB-2007	EU264561	*Plagiopyla nasuta*	Z29442
*Colpodidium caudatum*	EU264560	Pithites vorax*	FJ870070
*Condylostentor auriculatus*	DQ445605	*Prodiscophrya collini*	AY331802
*Discophrya collini*	L26446	*Prorodon teres*	X71140
Dysteria brasiliensis*	EU242512	*Prorodon viridis*	U97111
Dysteria derouxi*	AY378112	***Pseudochilodonopsis*** ** cf. ** ***fluviatilis***	JN867021
Dysteria procera*	DQ057347	*Pseudomicrothorax dubius*	FM201298
*Dysteria* sp. 1	AY331797	*Tokophrya lemnarum*	AY332720
*Dysteria* sp. 2	AY331800	*Tokophrya quadripartita*	AY102174
*Ephelota gemmeipara*	DQ834370	Trichopodiella faurei*	EU515792
Frontonia lynni*	DQ190463	Trithigmostoma cucullulus*	FJ998037
Frontonia tchibisovae*	DQ883820	*Trithigmostoma steini*	X71134
*Furgasonia blochmanni*	X65150	Trochilia petrani*	JN867016
Hartmannula derouxi*	AY378113	Trochilioides recta*	JN867017
***Heterohartmannula fangi*** *	FJ868204	Trochochilodon flavus*	JN867018
*Heliophrya erhardi*	AY007445	Uronychia setigera*	AF260120
Hypocoma acinetarum*	JN867019	Uronychia transfuga*	EF198669
*Isochona* sp.	AY242119	*Zosterodasys transverses*	EU286812
Leptopharynx costatus*	EU286811		

Species newly sequenced in the present study are marked in bold. Species sequenced by the authors' group are maked by sterisks (*).

A Bayesian inference (BI) was performed with MrBayes 3.1.2 [28] using the GTR+I+G evolutionary model indicated by MrModeltest v.2 [29]. The program was run for 1,000,000 generations with a sample frequency of 100 and a burn-in of 2,500. All trees remaining after discarding the burn-in were used in calculation of posterior probabilities using a majority rule consensus.

The program Modeltest 3.7 [30] selected GTR+I+G (dataset 1: G = 0.5422, I = 0.2922; dataset 2: G = 0.5628, I = 0.2835) under AIC criterion as the best model, which was then used for maximum likelihood (ML) analysis. A ML tree was constructed with the PhyML v2.4.4 program [31]. The reliability of internal branches was assessed using the non-parametric bootstrap method with 1,000 replicates.

A maximum parsimony (MP) tree was produced based on parsimony-informative sites (dataset 1: 655 sites; dataset 2: 648 sites) with PAUP* 4.0b10 [32]. The reliability of internal branches was estimated by bootstrapping with 1,000 replicates.

Seven constrained ML analyses were carried out by PAUP* 4.0b10 [32] according to the constraints listed in Table 2. Resulting constrained topologies were then compared to the non-constrained ML topology using the Approximately Unbiased (AU) test [33] as implemented in CONSEL v0.1 [34]. For all constraints, internal relationships within the constrained groups were unspecified, and relationships among the remaining taxa were unspecified as well.

**Table 2:** Approximately Unbiased (AU) test results.

Topology constraints	−Ln likelihood	AU value (*p*)
• unconstrained	15543.82506	0.982
1 *Chlamydodon* monophyletic	15561.89373	0.169
2 Chlamydontidae+Chilodonellide+Lynchellidae monophyletic	15577.48381	0.010
3 Chlamydontidae+Lynchellidae monophyletic	15577.74157	0.007
4 *Dysteria* monophyletic	15553.84383	0.189
5 *Pithites vorax*+*Trochochilodon flavus* monophyletic	15581.58673	0.002
6 *Pithites vorax*+*Trochochilodon flavus*+Hartmannulidae+Dyesteriidae monophyletic	15625.36216	0.002
7 *Microxysma acutum*+Hartmannulidae monophyletic	15595.58459	0.002

*p*<0.05 refute monophyly; *p*>0.05 do not refute the possibility of monophyly.

Results in which *p*<0.05 are marked in bold and shaded in grey.

### Nomenclatural acts

The electronic version of this document does not represent a published work according to the International Code of Zoological Nomenclature (ICZN), and hence the nomenclatural acts contained in the electronic version are not available under that Code from the electronic edition. Therefore, a separate edition of this document was produced by a method that assures numerous identical and durable copies, and those copies were simultaneously obtainable (from the publication date noted on the first page of this article) for the purpose of providing a public and permanent scientific record, in accordance with Article 8.1 of the Code. The separate print-only edition is available on request from PLoS by sending a request to PLoS ONE, 1160 Battery Street, Suite 100, San Francisco, CA 94111, USA along with a check for $10 (to cover printing and postage) payable to “Public Library of Science”.

In addition, this published work and the nomenclatural acts it contains have been registered in ZooBank, the proposed online registration system for the ICZN. The ZooBank LSIDs (Life Science Identifiers) can be resolved and the associated information viewed through any standard web browser by appending the LSID to the prefix “http://zoobank.org/”. The LSID for this publication is: Gao et al article in PLoS ONE: urn: lsid: zoobank.org: act: 68A7A13F-341B-4F85-A898-6A30D3391516.

## Results

### Phylogenetic trees

The topologies of all trees are generally consistent with the classification schemes proposed by previous researchers (Table S1). The class Phyllopharyngea is a monophyletic clade with four distinct groups, Cyrtophoria, Chonotrichia, Suctoria, and Rhynchodia. Cyrtophoria consists of two distinct groups: Dysteriida and Chlamydodontida, with Chonotrichia nested in Dysteriida (see Discussion below). Suctoria and Rhychodia are positioned as peripheral branches of Cyrtophoria, while the class Nassophorea is the nearest “out-group” to the class Phyllopharyngea. These results are also in agreement with previous reports [12], [13], [15], [16].

The order Dysteriida is a monophyletic clade, consisting of two well-separated groups, the families Dysteriidae and Hartmannulidae. Within Dysteriidae, *Mirodysteria* was always placed within the species of *Dysteria*. *Microxysma* clustered with *Trochilia*, rather than with species of Chlamydodontida as suggested by previous schemes (Table S1). Within Hartmannulidae, the newly sequenced *Aegyriana* grouped with *Trichopolliella*, which then clustered with *Hartmannula* and *Heterohartmannula*. *Trochiliodes* formed a basal branch out of the above four genera. Unlike the above two families, the branching order of Plesiotrichopidae was not unambiguously resolved in the present topologies. *Trochochilodon* always appeared as a peripheral branch out of Dysteriida (+Chonotrichia) (Figs. 2, S1, S2). However, the position of *Pithites* is uncertain; it clustered with species of Lynchellidae in the MP tree from dataset 2 (with low bootstrap value, Fig. S2), but branched outside of and parallel to Chlamydodontida in other trees (Figs. 2, S1).

The order Chlamydodontida was divided into three well-defined families, Chlamydodontidae, Chilodonellidae, and Lynchellidae. In the family of Chlamydodontidae, *Paracyrtophoron* is nesting within *Chlamydodon*. On the other hand, the topology of the family Chilodonellidae is congruent with previous schemes (Table S1), within which *Pseudochilodonopsis* formed a clade with *Chilodonella* and further clustered to two species of *Trithigmostoma*. In the family of Lynchellidae, four genera, *Chlamydonella*, *Chlamydonellopsis*, *Lynchella*, and *Coeloperix*, were sequenced for the first time and analyzed in the present work. They formed consistently a monophyletic clade in all topologies, and thus correspond to the concept of the family Lynchellidae according to Jankowski [35]. Within these four genera, two groups were recognized; one is *Chlamydonella* and *Chlamydonellopsis*, and the other is *Lynchella* and *Coeloperix*. The close relationship of *Coeloperix and Lynchella* is a true reflection of their similar morphology with a slight difference (presence of CSB in *Lynchella* vs. absence in *Coeloperix*) [36].

A species of Chonotrichia, *Isochona* sp., grouped with harmannulids, while the only sequenced genera of Rhynchodia, *Hypocoma*, formed a sister clade with the monophyletic clade of Suctoria which branches basally from all cyrtophorians (Figs. 2, S1, S2).

### Analyses of introns in the SS rRNA gene of *Aegyriana oliva*

**Figure 3:**
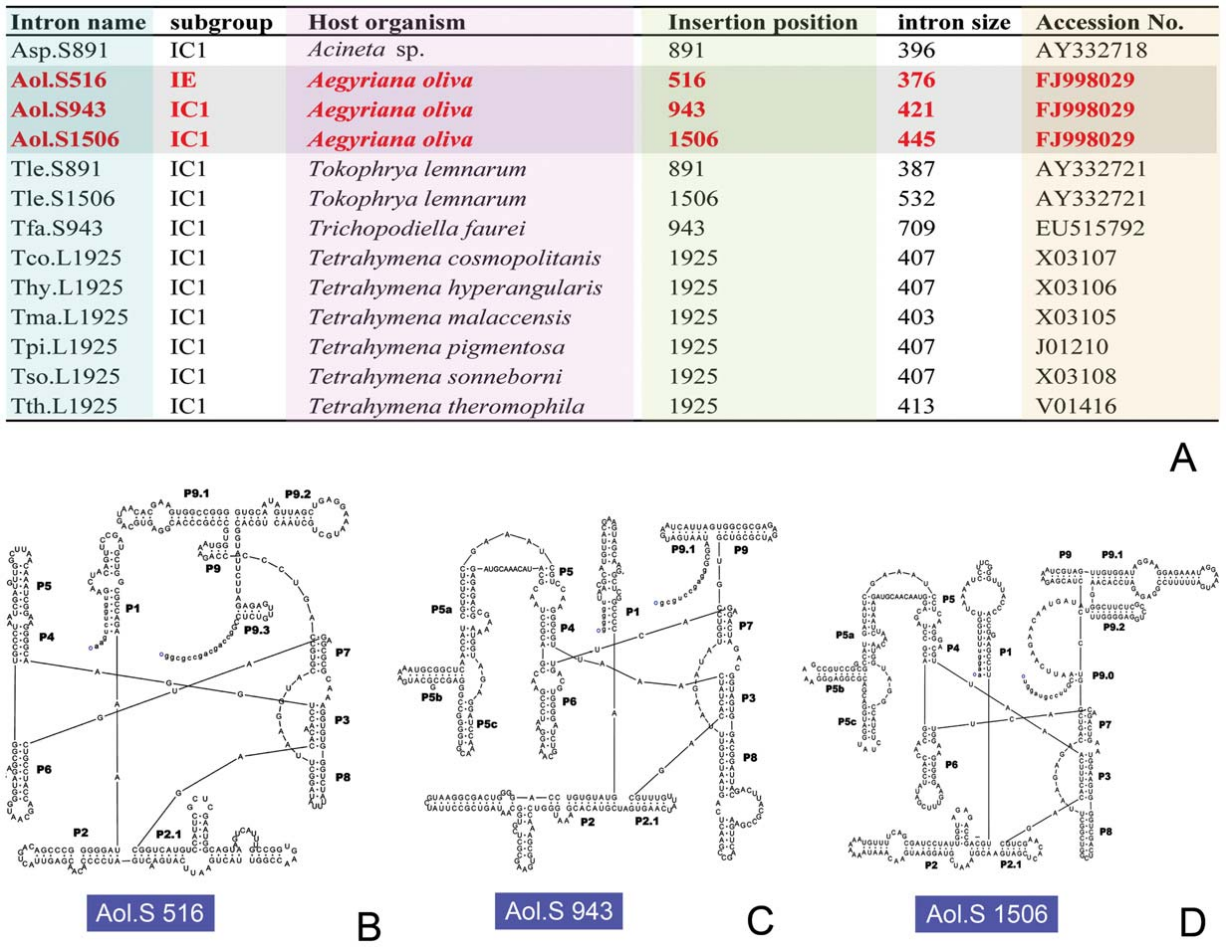
Three group I introns in the small subunit ribosomal RNA gene of *Aegyriana oliva*. **A.** Summary of reported group I introns in ciliates. The species reported in the present study are marked in bold. **B–D.** Secondary structure of three introns predicted by the GISSD database. **B.** Aol. S516. **C.** Aol. S943. **D.** Aol. S1506.

We discovered three group I introns (376–446 nucleotides) in the SS rRNA gene of *Aegyriana oliva* (Fig. 3). They are at position 516, 943, and 1506 of the SS rRNA gene of *E. coli* (J01695), which are named as Aol.S516, Aol.S943, and Aol.S1506 following Johansen and Haugen [37]. The predicted secondary structure showed that Aol.S516 was affiliated with the IE1 group, while Aol.S943 and Aol.S1506 were affiliated with the IC1 group (Figs. 3B–3D).

## Discussion

### The order Chlamydodontida is a paraphyly

Even though all of the three constituent families were monophyletic groups, our results consistently showed that the order Chlamydodontida was a paraphyletic assemblage. Moreover, the AU test in this study, with an expanded set of sequences (10 genera, 13 species), refuted the possibility that Chlamydodontida is a monophyletic clade (Table 2, constraint 2, *p* = 0.01) and confirmed the reliability of phylogenetic results. This is in concerto with other studies, even though only four species were included in previous molecular trees [12], [13], [15], [16].

Based on the ciliary patterns and the structure of macronucleus, Gong [19] assigned the families with juxtaposed heteromerous macronucleus, Chilodonellidae and Lynchellidae, into the suborder Chlamydodontina, while placed Chlamydodontidae (+Gastronautidae) with centric heteromerous macronucleus into Chilodonellina. This assignment agrees with the scheme proposed by de Puytorac [4] (Table S1), but was not supported by our phylogenetic results, in which these three families formed separate monophyletic clades. Accordingly, the AU test rejected the possibility that Chilodonellidae and Lynchellidae belong to a monophyletic group (Table 2, constraint 3, *p* = 0.007), suggesting that the feature of macronucleus may not be a strong diagnostic character to distinguish monophyletic groups.

### The relationship between *Paracyrtophoron* and *Chlamydodon*

In our analyses, *Paracyrtophoron* nested within the species of *Chlamydodon*. However, *Paracyrtophoron* can be easily distinguished from *Chlamydodon* by the lack of the cross-striped band (CSB) around the periphery of the somatic field [38]. Such discrepancies could be attributed to an evolutionary scenario that the CSB is a convergent character with some members of the Lynchellidae, which may not be reflected in the SS rRNA sequences. Moreover, the AU test did not refute the possibility that *Chlamydodon* is a monophyletic clade (Table 2, constraint 1, *p* = 0.169). At this point, the available evidence could not support the paraphyly of *Chlamydodon*.

#### *Microxysma* is a member of the family Dysteriidae

The major features to distinguish Hartmannulidae and Dysteriidae are the body shape and the structure of left ventral kineties [5]. In Hartmannulidae, the body is conspicuously dorsoventrally flattened, and the left ventral kineties are generally developed and continuous with the right ones, whereas in Dysteriidae, the body is mostly highly bilaterally flattened with the left kineties extremely reduced and restricted to the equatorial area [9]–[11].

In all previous morphology-based classification schemes (Table S1), *Microxysma* was arranged in the family Hartmannulidae. But this assignment is not supported by our molecular trees, in which *Microxysma* was placed away from the species of Hartmannulidae. Moreover, the possibility that *Microxysma* and species of Hartmannulidae are monophyletic was also refuted by the AU test (Table 2, constraint 7, *p* = 0.002). In fact, there is a large morphological difference between *Microxysma* and hartmannulids. In *Microxysma*, the highly shortened left kineties were degenerate to a limited area, which are practically different from those in the typical hartmannulid species, whose kineties cover the majority of the left side. Rather, the bilaterally compressed *Microxysma* shares the basic pattern of ciliature with the species in Dysteriidae, e. g. right kineties are arranged along the narrow ventral margin with the reduced left field of kineties [19]. Compared with other typical dysteriids, the ciliary pattern of *Microxysma* is similar to that of the dysteriid *Trochilia*, which can explain its neighboring position to the latter in all topologies of the molecular trees. Therefore, both morphological and molecular data suggest that *Microxysma* should be transferred from Hartmannulidae to Dysteriidae.

### The paraphyly of the family Plesiotrichopidae and the systematic positions of *Trochochilodon* and *Pithites*, with establishment of a new family Pithitidae n. fam

The family Plesiotrichopidae was erected by Deroux [8], diagnosed roughly by having “*Chilodonella*-like infraciliature and adhesive apparatus located centrally in ventral depression”. As shown in Table S1, Plesiotrichopidae was tentatively assigned into the order Dysteriida in most classification schemes [1], [2], [4], [5], [9], however, up to date, the relationships/systematic positions of taxa in this family have never been investigated using molecular information. We supplemented the knowledge by analyzing the phylogeny of this family based on the SS rRNA gene sequence data of two genera, *Trochochilodon* and *Pithites*. It indicates that the two genera are systematically far away from each other, rendering the family Plesiotrichopidae a paraphyletic assemblage. These results correspond well to the morphological and morphogenetic dissimilarities between the two genera: both the structure of buccal apparatus and the formation process during the binary fission are considerably different from each other [8], [18], [39]. The topology also suggests that neither of them should be placed in the current order Dysteriida, because *Trochochilodon* grouped outside the order Dysteriida, while *Pithites* located basally to the other cyrtophorians. Therefore, both the molecular and the morphological/morphogenetic data challenge the scheme to arrange them in the same family.

Unfortunately, the systematic position and the definition of the family Plesiotrichopidae still remain unsolved at the present stage. The problem is that the molecular data for the type genus *Plesiotrichopus* are totally lacking and not many taxonomic characters can be used to characterize genera within the family. As a result, few pieces of evidence are available to define which one is near to the type genus. Another confusion comes from the presence of a dominant tube-like structure (secretory channels) in *Plesiotrichopus*, which is absent in *Pithites* and *Trochochilodon*. If it is a critical feature of this family, both *Pithites* and *Trochochilodon* should be transferred from the current taxon. Currently, the family Plesiotrichopidae is an *incertae sedis* taxon.

Regarding the phylogeny, no close relationship between *Pithites* and dysteriids was recovered. Moreover, the possibility that *Pithites* and Dysteriidae form a monophyletic clade was also rejected by the AU test (Table 2, constraint 6, *p* = 0.002), which is also supported by the morphological features. For example, taxa in the order Dysteriida are diagnosed by the presence of the adhesive organelle (typically a flexible podite) that is absent in Chlamydodontida [1], [2], [5], [8], [39], whereas *Pithites* has no such organelle. Even though a filament from the secretary channel (character of *Plesiotrichopus*) was mentioned in *Pithites* by Deroux and Dragesco [40], it is not confirmed in the *in vivo* observations by Pan [18]. In addition, *Pithites* has separated left and right kineties which is never seen in dysteriids (vs. continuous). Given that *Pithites* has a peripheral position to Chlamydodontida in most topologies, lacks the podite and possesses a unique oral structure (apically located, several kinety fragments radiated around the cytostome), it may belong to an isolated taxon (at least) at family level and should be moved from Dysteriida to the order Chlamydodontida. Therefore, we suggest a new family here, Pithitidae n. fam. with the type genus *Pithites*, under the order Chlamydodontida (urn: lsid: zoobank.org: act: 68A7A13F-341B-4F85-A898-6A30D3391516). The family is characterized by the combination of the following features: (1) pelagic forms with almost non-compressed body shape and apically positioned cytosotme; (2) well developed somatic kineties on both left and right fields with a conspicuous cilia-free area between them; (3) oral apparatus consisting of several kinety fragments around the cytostome; and (4) without podite but having a “thigmotactic field” subcaudally near the meridian of ventral side where the thread-like adhesive organelle is located [39].

Meanwhile, it is relatively certain that the genus *Trochochilodon* should also be transferred from Dysteriida to Chlamydodontida. According to the observations by Pan [41], this *Chilodonella*-like taxon is very similar to chlamydodontid species. The former differs from the latter only by having two preoral kineties (vs. mostly three in chlamydodontids) and the cilia-free field between left and right somatic kineties is inconspicuous (dominant in some chlamydodontids; Fig. 1). Regarding the position revealed in our SS rRNA-based topological analyses, it is reasonable to deduce that this organism might represent an intermediate form closer to chlamydodontids than to dysteriids [8]. However, whether it belongs to the family Plesiotrichopidae still needs further explorations, because the molecular information of the type genus *Plesiotrichopus* is currently lacking.

In summary, three conclusions can be drawn: (1) the current family Plesiotrichopidae consists of paraphyletic clades and most of them are systematically unclear; (2) both *Pithites* and *Trochochilodon* should be transferred from the order Dysteriida, and they likely belong to Chlamydodontida; and (3) based on both morphological/morphogenetic and molecular information, a new family, Pithitidae n. fam. is suggested for the genus *Pithites*.

### Data of *Isochona* sp. might come from a misidentified organism

*Isochona* sp., the only sequenced species of the subclass Chonotrichia, was positioned basally to other hartmannulids in our results. However, morphologically, chonotrichians are a highly specialized group with numerous unique characters, e.g. the attaching living style (or aufwuchs) with flask-shaped body, non-fused conjugation process, and highly reduced infraciliature which is spirally arranged and limited within the choler wall, etc. [2], [11]. All the above criteria indicate that they should be clearly distinguished from the taxa of cyrtophorians. A reasonable explanation for our phylogenetic result is that the material was misidentified. Species in Chonotrichia are un-cultivatable and, as periphyton forms, they are easily mixed with other attaching ciliates when sampled. Moreover, only one population/species (*Isochona* sp.) from this subclass has been sequenced so far. Thus, the sequence submitted to the GenBank database is likely from a misidentified organism, that is, a cyrtophorid instead of a chonotrich.

### Fine-scale investigation of the order Dysteriida

As stated above, *Pithites* and *Trochochilodon* were transferred from the order Dysteriida to Chlamydodontida, and *Isochona* is likely to be a hartmannulid. This leaves the order Dysteriida as a monophyletic clade, with two well-supported groups, Dysteriidae and Hartmannulidae. The clear separation of these two families was expected on the basis of their distinguished morphology: species in Dysteriidae have “left ventral somatic kineties as midventral postoral field, typically separated from an anterior preoral field”, and those in Hartmannulidae have “left ventral somatic kineties, which may be quite short, as continuous field” [5].

In addition, Dysteriidae and Hartmannulidae are revealed as closely related sister group (Fig. 2, BI/ML:1.00/92), and they both share a very similar secondary structure of the V2 region. This corresponds to the fact that they both embrace the ordinal character such as dorsoventrally compressed body shape, non-thigmotactic ventral cilia, and juxtaposed heteromerous macronucleus [5].

### Group I introns in cyrtophorids

Four group I introns have been reported in the SS rRNA gene of three ciliates, with two in *Tokophrya lemnarum*, and one in *Acineta* sp. and *Trichopodiella faurei* each [12], [13]. In our current work, *Aegyriana oliva* is the fourth reported ciliate embracing introns, and is also the first reported ciliate having three introns, namely Aol.S516, Aol.S943, and Aol.S1506. The S943 was first reported in *Trichopodiella faurei* [12], while the S1506 intron was only described in *Tokophrya lemnarum* [13]. The Aol.S516, to our knowledge, is the first intron reported at position 516 of the ciliate SS rRNA gene.

On the basis of the conserved secondary structure, conserved core nucleotide regions, and phylogenetic analysis, group I introns have been classified into five major groups: IA, IB, IC, ID, and IE [42]. Aol.S943 and Aol.S1506 belong to the IC group, as well as the four previously reported SS rRNA introns and nine LS rRNA introns. By contrast, Aol.S516 is the only IE group I intron discovered in ciliates so far (Fig. 3A). Interestingly, all the above species embracing SS rRNA introns belong to the class Phyllopharyngea, while LS rRNA introns were only reported in the tetrahymenid genus *Tetrahymena* (Fig. 3A), which belongs to the class Oligohymenophorea, a group far away from the cyrtophorians [1]. Regarding the different structural features and scattered systematic positions of those introns, it is still too premature to evaluate their evolutionary significance.

## Supporting Information

Figure S1 Phylogenetic trees inferred from small subunit rRNA gene sequences (dataset 1) with an emphasis on cyrtophorid ciliates. Numbers on branches are the following: bootstrap values from maximum likelihood (ML) analysis, followed by the Bayesian posterior probability value and the bootstrap values of maximum parsimony (MP) analysis. Solid circles represent full bootstrap support in all three algorithms and hyphen (-) represents support values below 0.50/50%. Species sequenced in the present study are shown in bold.
(TIF)

Figure S2 A maximum-parsimony tree inferred from the small subunit ribosomal RNA gene sequences (dataset 2). Species sequenced in this work are marked in bold. Numbers at the nodes represent the bootstrap values.
(TIF)

Table S1 Taxonomic schemes for the classification of cyrtophorid ciliates. Species newly sequenced in the present study are in grey.
(XLS)

Table S2 Sampling sites and habitat information of species sequenced in this study.
(XLSX)
