# Supplementary figures and images for: Molecular Phylogeny of the Cyrtophorid Ciliates (Protozoa, Ciliophora, Phyllopharyngea)
Source: PLoS One. 2012 Mar 12;7(3):e33198. doi: 10.1371/journal.pone.0033198 (PMC3299757; doi:10.1371/journal.pone.0033198)

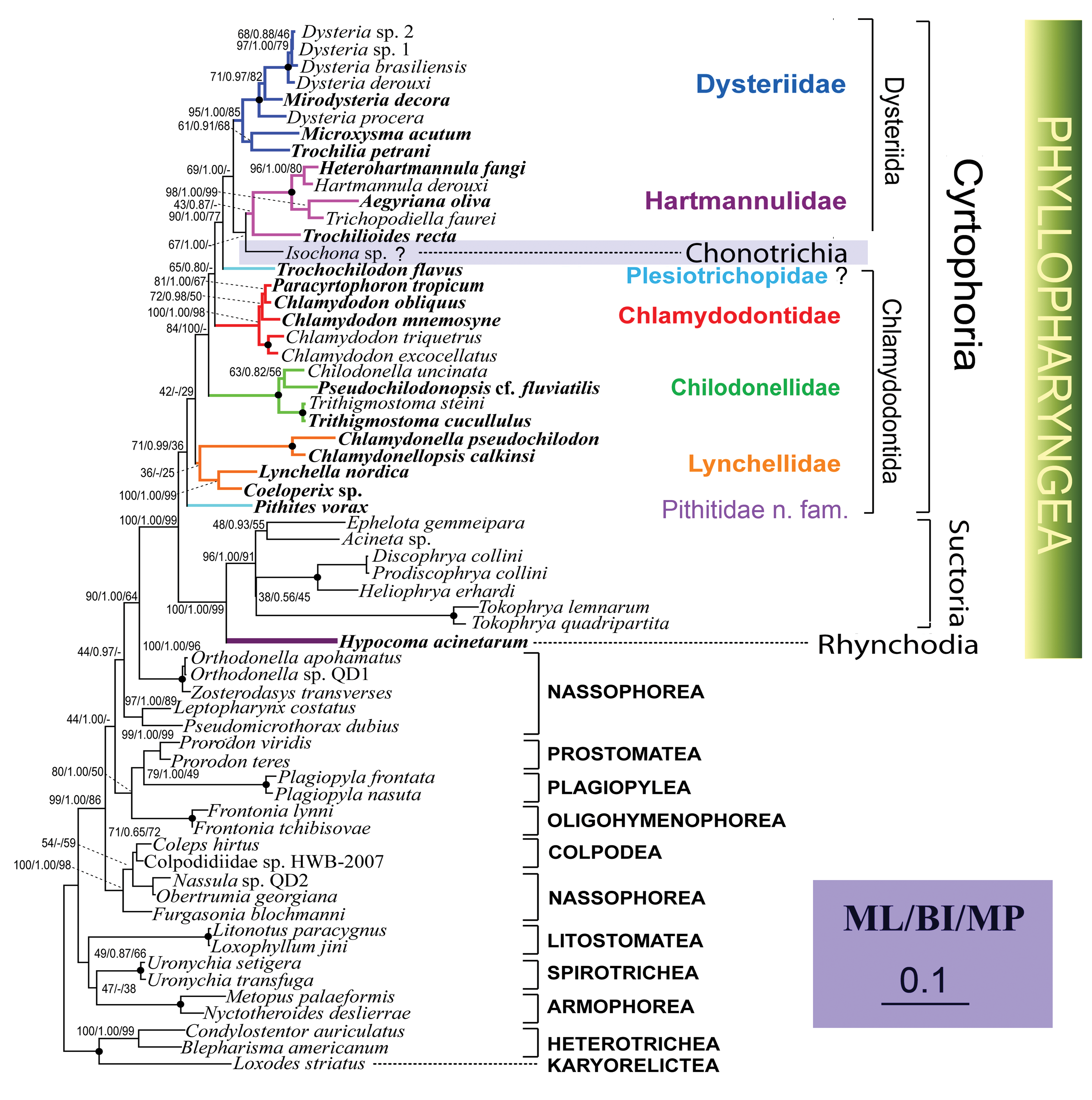

Supplement: Figure S1 — Phylogenetic trees inferred from small subunit rRNA gene sequences (dataset 1) with an emphasis on cyrtophorid ciliates. Numbers on branches are the following: bootstrap values from maximum likelihood (ML) analysis, followed by the Bayesian posterior probability value and the bootstrap values of maximum parsimony (MP) analysis. Solid circles represent full bootstrap support in all three algorithms and hyphen (-) represents support values below 0.50/50%. Species sequenced in the present study are shown in bold. (TIF) [file pone.0033198.s001.tif]

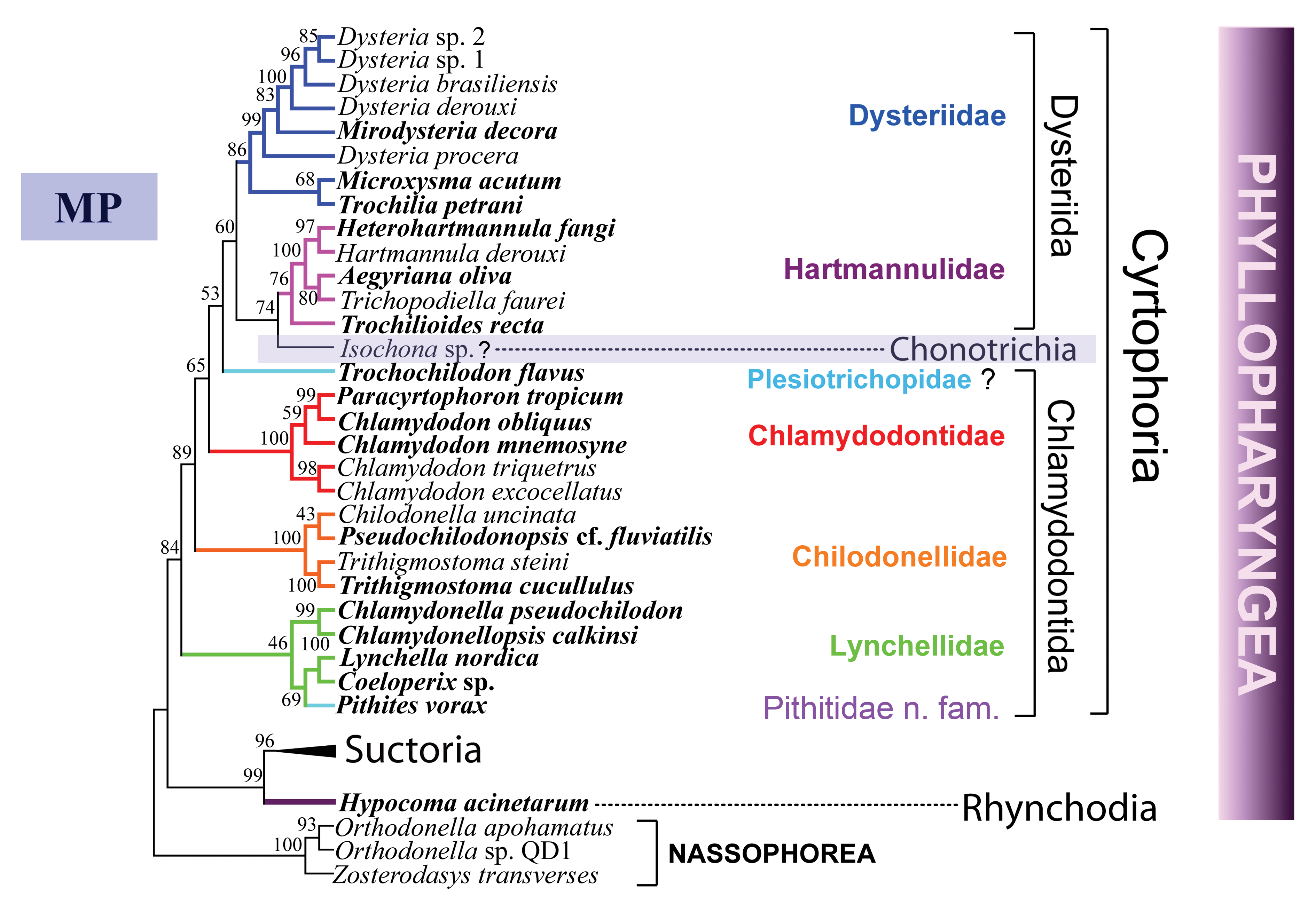

Supplement: Figure S2 — A maximum-parsimony tree inferred from the small subunit ribosomal RNA gene sequences (dataset 2). Species sequenced in this work are marked in bold. Numbers at the nodes represent the bootstrap values. (TIF) [file pone.0033198.s002.tif]
